# CrowdRadar: a mobile crowdsensing framework for urban traffic green travel safety risk assessment

**DOI:** 10.3389/fdata.2025.1440816

**Published:** 2025-03-21

**Authors:** Yigao Wang, Qingxian Tang, Wenxuan Wei, Chenhui Yang, Dingqi Yang, Cheng Wang, Liang Xu, Longbiao Chen

**Affiliations:** ^1^Fujian Key Laboratory of Sensing and Computing for Smart Cities (SCSC), School of Informatics, Xiamen University, Xiamen, Fujian, China; ^2^Department of Computer and Information Science/State Key Laboratory of Internet of Things for Smart City, University of Macau, Taipa, Macau SAR, China; ^3^Artificial Intelligence Innovation Center, Yangtze Delta Region Institute of Tsinghua University, Jiaxing, Zhejiang, China

**Keywords:** Urban traffic, traffic data, mobile crowdsensing, urban computing, computer vision

## Abstract

As environmental awareness increased due to the surge in greenhouse gases, green travel modes such as bicycles and walking have gradually became popular choices. However, the current traffic environment has many hidden problems that endanger the personal safety of traffic participants and hinder the development of green travel. Traditional methods, such as identifying risky locations after traffic accidents, suffer from the disadvantages of delayed response and lack of foresight. Against this background, we proposed a mobile edge crowdsensing framework to dynamically assess urban traffic green travel safety risks. Specifically, a large number of mobile devices were used to sense the road environment, from which a semantic detection framework detected the traffic high-risk behaviors of traffic participants. Then multi-source and heterogeneous urban crowdsensing data were used to model the travel safety risk to achieve a comprehensive and real-time assessment of urban green travel safety. We evaluated our method by leveraging real-world datasets collected from Xiamen Island. Results showed that our framework could accurately detect traffic high-risk behaviors with average F1-scores of 86.5% and assessed the travel safety risk with *R*^2^ of 0.85 outperforming various baseline methods.

## 1 Introduction

The rapid industrialization and urbanization worldwide have led to a significant increase in greenhouse gas emissions, exacerbating climate change. The Intergovernmental Panel on Climate Change (IPCC) predicted that global CO2 emissions must decrease by about 45% from 2010 levels by 2030 to limit global warming to 1.5°C. At the same time, the transportation sector, a major source of carbon emissions, has seen the growing popularity of green travel modes such as cycling and walking in recent years, driven by increasing awareness of emission reduction (Jia et al., 2017). However, the current situation of urban traffic may not fully meet the safety needs of green travel. For example, in some large cities in China, the safety issues of cyclists and pedestrians have become particularly prominent: the risk of traffic accidents for cyclists who run red lights is more than 3 times that of those who never run red lights; the risk of accidents for cyclists carrying adults is more than 2 times that of those who never carry adults; the risk of riding on the motorway is 2.4 times that of normal riding (Qian et al., 2020). These hidden dangers not only endanger the personal safety of traffic participants, but also hinder the development of low-carbon transformation and green travel. Therefore, urban management departments need a method to evaluate the safety of the urban traffic environment in order to optimize the urban traffic environment and achieve green travel.

However, assessing the safety of urban traffic green travel is not trivial. Traditionally, the urban authorities identified high-risk hotspots dependent on human experiences or historical accidents datasets and then sent volunteers to persuade or put traffic signs to warn traffic participants with weak safety awareness. However, the method is hard to implemented in real-time which consumes a great amount of labor and time. In addition, the authorities usually identify what needs to be rectified after the occurrence of traffic accidents, which suffers from hysteresis and blindness. At the same time, many scholars have tried to improve traffic safety assessment and high-risk behavior detection through mobile crowd sensing (MCS) technology. For example, existing research used smartphones and IoT devices to collect traffic data, mainly focusing on traffic flow monitoring, environmental perception and other fields (Ganti et al., 2011). Although these methods have achieved certain results in traffic flow prediction and environmental monitoring, they often ignore the real-time monitoring of traffic participants' behaviors, especially high-risk behaviors. Existing high-risk behavior detection methods mostly rely on fixed cameras or specific scenes, which are difficult to adapt to the complexities and dynamic changes of urban traffic (Ahmed et al., 2019). This research gap makes it difficult for existing technologies to discover and predict high-risk behaviors that may lead to traffic accidents in real time. In addition, in the field of traffic accident risk assessment, many traditional models rely on historical data to predict the likelihood of accidents. These methods usually ignore the immediate changes in the traffic environment (de Medrano and Aznarte, 2021). Therefore, these methods have lags and cannot respond to changes in traffic conditions in a timely manner. In practical applications, it is difficult to deal with sudden high-risk events. Therefore, a low-cost, real-time, and comprehensive method for transportation safety assessment and decision making is in demand.

Fortunately, the rapid development of sensor-rich mobile edge devices have generated large-scale public area images and promoted a mobile-user-centric crowdsensing paradigm for sensing data (Lee and Lee, 2015). In addition, the remarkable progress of the Internet of Things, computer vision, and deep learning technologies in recent years has made it possible to efficiently understand traffic behaviors through images and video streams in the edge or cloud (Zou et al., 2023; Buch et al., 2011; González et al., 2011). These mobile edge devices and deep learning-based algorithms provide us with an unprecedented opportunity for automatically assessing the safety risk of traffic green travel on an urban scale.

In this work, we propose CrowdRadar, a crowdsensing-based framework to assess the safety risk of urban traffic green travel leveraging mobile edge devices, deep learning-based traffic high-risk behavior detection method, and an urban crowdsensing data-based travel risk assessment model. Specifically, we firstly used a large number of mobile edge devices to sense the road environment. Then we detected the high-risk behaviors of traffic participants and assess the risk of the road environment according to urban crowdsensing data, to achieve a comprehensive and real-time assessment of urban traffic green travel safety.

In designing the framework, there are several research issues to be addressed:

- **It is not trivial to efficiently collect road sensing data in a privacy perserving manner**. Intuitively, we can require mobile edge devices to transmit video or multimedia streams to the cloud center in real-time. However, simply using the mobile network to upload such a large amount of data may cause inconvenience to mobile phone users, resulting in additional cellular data costs and heavy battery consumption (Lane et al., 2013). In addition, the raw data collected through the visual crowdsensing paradigm would expose users' privacy, including participant and third-person (Guo et al., 2017). Therefore, an edge device is needed to solve multimedia data preprocessing and privacy protection problems.

- **It is difficult to accurately identify traffic high-risk behaviors in a low-cost manner**. Traditionally, we need to train an identification model for each traffic high-risk behavior. For example, a GoogleNet model is trained to classify whether a motorcyclist is wearing a helmet or not after the motorcyclist is detected through YOLO (Chairat et al., 2020). However, the traditional method is difficult to expand and adapt to diverse traffic behaviors because it requires a large amount of training data for each type of behavior. Therefore, we need an accurate, semantic general detection framework to identify different traffic high-risk behaviors and a low-cost data annotation method.

- **It is essential to assess travel safety risks with urban crowdsensing data**. The safety risk assessment of urban traffic green travel is usually complicated and closely related to many factors, such as the high-risk behaviors of traffic participants or truck trajectories. It is difficult to assess the safety only by road sensing data. Therefore, multi-source and heterogeneous urban crowdsensing data should be further introduced to jointly establish the safety assessment model.

To address these issues, we proposed an edge-cloud hybrid crowdsensing framework to assess urban traffic green travel safety risk. Firstly, we developed an edge device with multiple sensors based on the Nvidia computing platform to collect the road sensing data. The edge device is equipped with a real-time object detection algorithm to transmit the regions of interest (ROI) to the cloud center for reducing network transmission consumption. Additionally, the device includes a privacy-preserving module to blur pedestrians' faces and license plates, ensuring the protection of users' privacy.

Secondly, to accurately and low-cost identify traffic high-risk behaviors, we proposed a semantic detection framework that requires a small amount of labor cost. Specifically, we first fine-tuned the object detection model (Varghese and Sambath, 2024) and the relation detection model (Gao et al., 2018) to detect the location and the relation of traffic participation objects. Then, an unsupervised learning graph-embedding algorithm was used to transfer the traffic graph, which consists of the location and relation of traffic objects, to a graph embedding. Finally, a lightweight classification model was used to classify these graph embedding to traffic high-risk behaviors.

Thirdly, to assess the safety risk of urban traffic green travel, we collected multi-source and heterogeneous urban crowdsensing data related to the travel environment, including traffic high-risk behaviors, truck trajectories, urban point of interest (POI), etc. Finally, we used a convolutional neural network (CNN) to extract the spatial features of the datasets outlined previously to assess environmental safety. In summary, the main contributions of this paper include:

- **Innovative framework design**: we propose CrowdRadar, a framework based on mobile edge devices and crowdsensing, dedicated to the safety assessment of urban traffic green travel. Compared to traditional methods, this framework offers significant advantages in terms of low cost, real-time performance, and comprehensiveness. It is particularly suitable for the complex and dynamic urban traffic environment, filling the gap where existing methods fail to address real-time high-risk behavior detection and comprehensive traffic safety assessment.

- **Real-time high-risk behavior detection and assessment**: we designed a three-phase framework that enables efficient and low-cost detection of high-risk traffic behaviors through a semantic general detection framework based on multiple deep learning models and an information retrieval-based data annotation module. Additionally, the framework performs comprehensive safety risk assessment using multi-source and heterogeneous urban crowdsensing data. This approach overcomes the limitations of traditional methods and provides a dynamic and scalable solution.

- **Empirical validation and performance improvement**: we evaluated the proposed framework using a real-world dataset from Xiamen. The results show that the framework can accurately detect high-risk traffic behaviors, achieving an average F1-score of 86.5%, and assess travel safety risks with an *R*^2^ value of 0.85, significantly outperforming various baseline methods. This demonstrates that our framework not only outperforms traditional methods in terms of accuracy but also has strong practical application potential.

## 2 Preliminaries and framework

**Definition 1**. ***Green Travel***: Green travel is derived from the concept of green transport, including walking, riding a bicycle, taking a bus, taking the subway, etc. (Yang et al., 2017). In this research, we mainly focus on human-centered green travel modes, such as walking and riding a two-wheeler.

**Definition 2**. ***Traffic High-risk Behaviors***: This paper focuses mainly on the daily traffic behavior of urban residents during green travel. Traffic high-risk behavior refers to actions that occur while walking or riding a bike and are likely to lead to traffic accidents, such as riding without wearing a helmet or running a red light, as detailed in Section 6.

**Definition 3**. ***Traffic High-risk Hotspots***: Traffic high-risk hotspots refer to locations with relatively more traffic accidents compared to other locations on the road network. These locations might become traffic hotspots due to traffic high-risk behaviors or other environmental factors.

**Figure 1:**
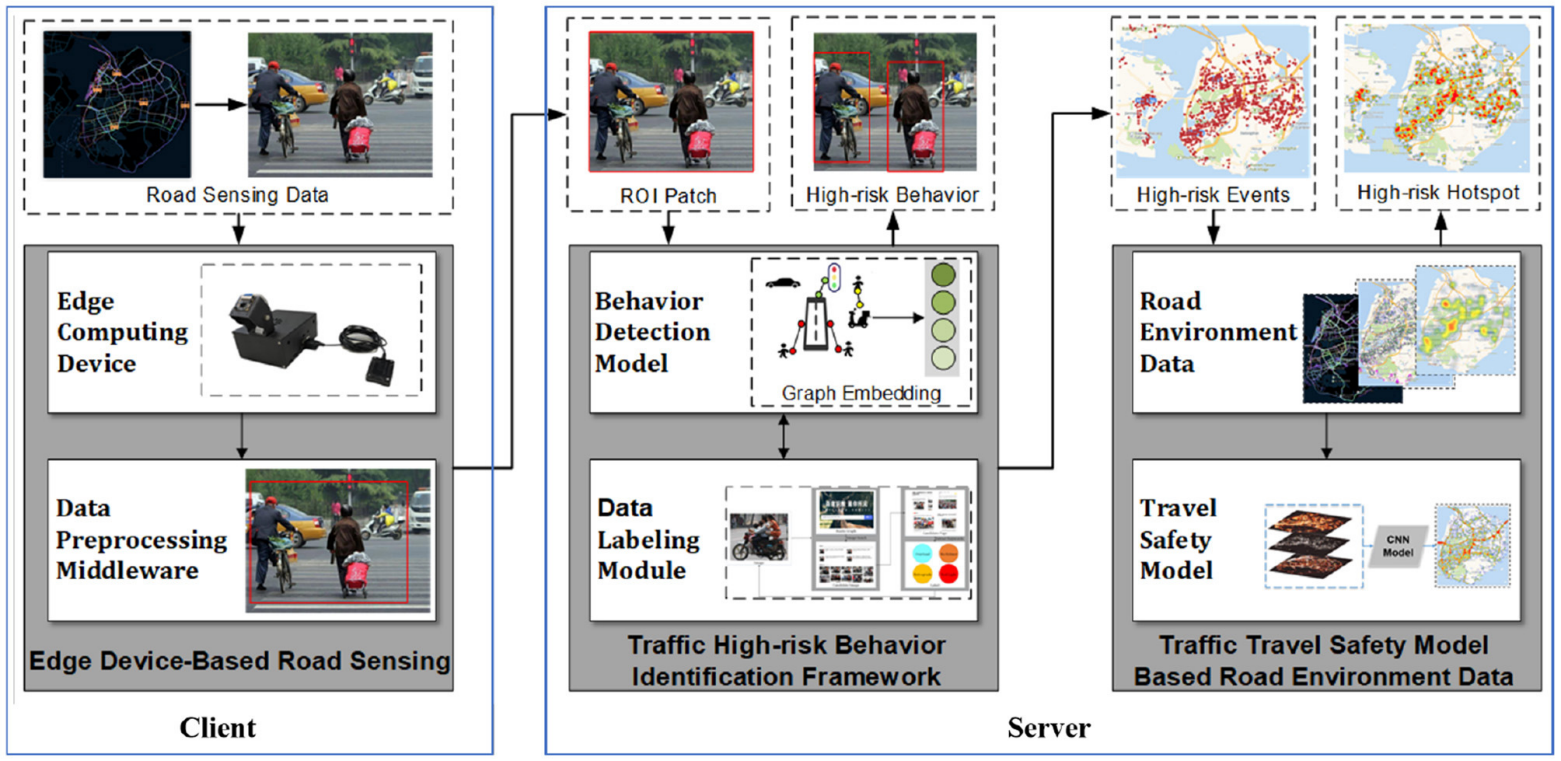
Framework overview.

We proposed a mobile edge crowdsensing framework to dynamically assess urban traffic green travel safety. As shown in Figure 1, we first developed an edge computing device to collect road perception data within the crowdsensing paradigm, employing object detection and relationship detection algorithms to identify traffic participants and the semantic relationships between them. This enabled the construction of a semantic traffic graph from traffic images. Simultaneously, ROI extraction, privacy protection, and other data preprocessing steps were performed on the edge device to ensure data validity and security. Subsequently, an unsupervised graph embedding algorithm was utilized to transform the semantic traffic graph into traffic graph vectors, facilitating the identification of high-risk traffic behaviors from multimedia data streams and generating labeled data for model training. Finally, leveraging multi-source heterogeneous urban crowdsensing data, we constructed a CNN model to assess the safety of green urban transportation. Additionally, this paper proposed a traffic image data annotation method based on information retrieval, utilizing a visual information retrieval platform for cost-effective and efficient data labeling. Supported by the high-risk traffic behavior recognition module, the framework generates high-risk traffic behavior distributions with spatial location information, providing robust support for urban traffic management.

## 3 Edge device-based road sensing

In this section, our goal is to efficiently collect road sensing data in a privacy-preserving manner. The traditional method of transporting multimedia streams results in some issues including large amounts of cellular network consumption, the waste of computing resources in cloud centers, and privacy-preserving concerns. To solve these issues, we developed an edge device-based road sensing platform which contained multiple sensors and data preprocessing modules to solve these issues.

### 3.1 Edge computing device

**Figure 2:**
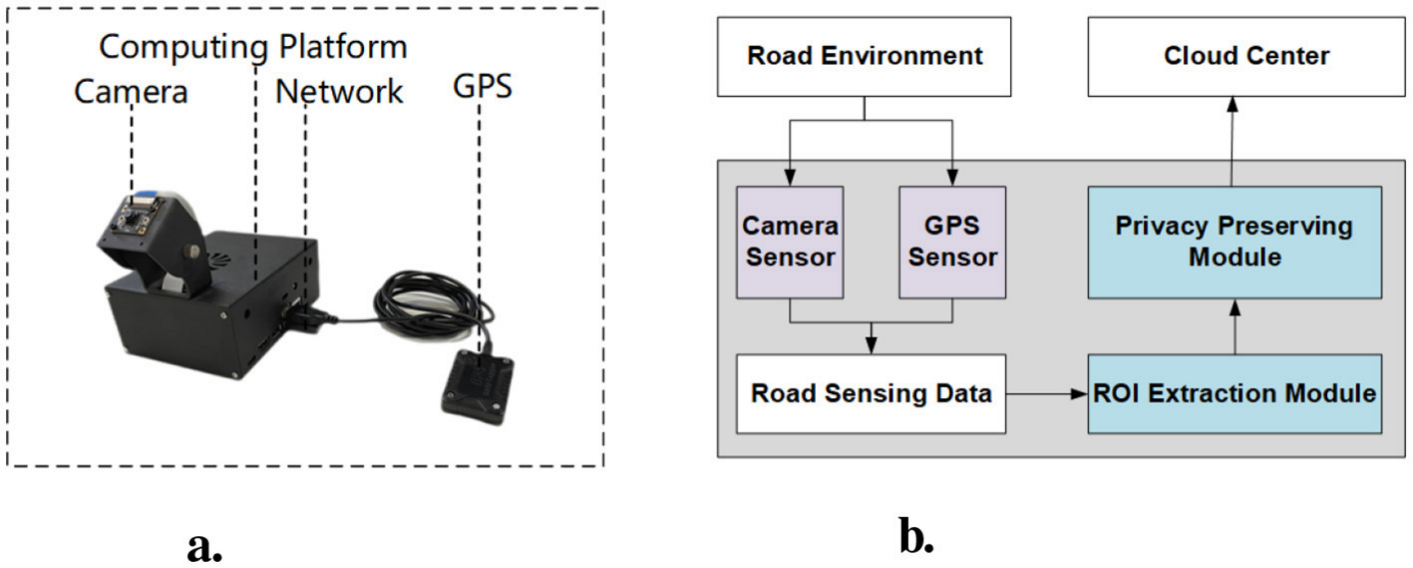
Visual comparisons of original models. **(A)** Hardware details. **(B)** Software architecture.

We use Nvidia Jetson Nano, a small and powerful computer for embedded applications and AIoT, as the embedded computing center. Based on Jetson Nano, we have adapted it with a compact case and a variety of sensors. As shown in Figure 2A, “Edge Box” has a camera, network and GPS module. We use a MIPI CSI-2 camera because it is small and easily fixed to the case. Considering the complex outdoor situation, we use a wireless network module and a GPS module with a USB extension cable. In addition, we can even use the common mobile power supply of mobile phones to power it, so the “edge box” can not only be placed on the vehicle as a mobile sensing device but also can be used as a fixed sensing device like an automated camera. Furthermore, Figure 2B demonstrates how edge devices collect data from the road environment and transmit it to the cloud center through computation.

### 3.2 Data preprocessing middleware

In this section, we introduce in detail how we use the edge device to achieve efficient collection of road sensing data under privacy protection.

#### 3.2.1 ROI extraction module

**Figure 3:**
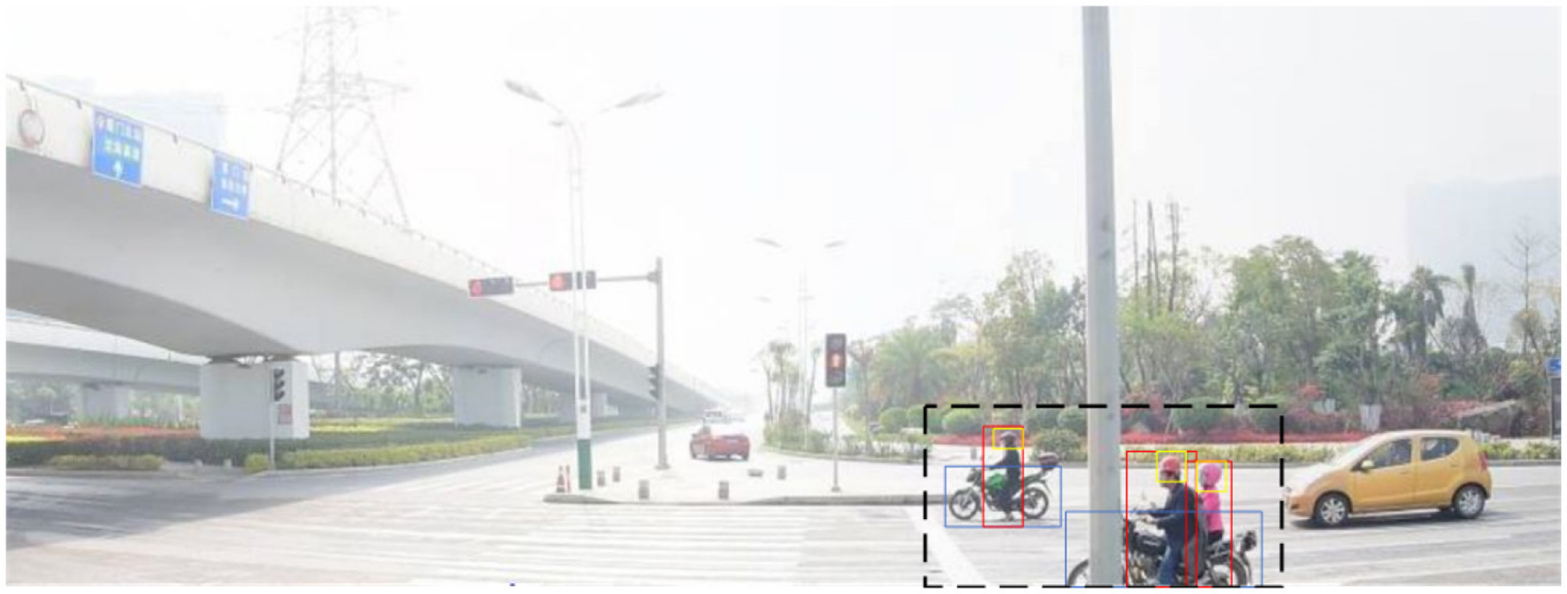
An illustration of ROI extraction result. The area surrounded by the black dashed boxes of the image should be cut and uploaded.

Traditionally, automated cameras are usually deployed at intersections to analyze road scenes (He et al., 2015). For those traffic systems that use complex algorithms in the cloud to process data (Shirazi and Morris, 2016), automated cameras need to transmit large amounts of data to the cloud (Leduc et al., 2008). To solve the problem of high communication cost (Skordylis and Trigoni, 2011), the edge devices need to have the computing capability to extract traffic behavior related-data. Figure 3 illustrates the process of extracting traffic behavior.

In this research, we focus on traffic high-risk behaviors in specific scenarios, one of which is human involvement. Intuitively, we might delete those areas with no human activity, remaining only the ROI patch. However, pedestrians might be distributed in various regions of the image in a densely populated road environment, which would cause difficulties for us to extract the ROI patch. Due to limited computing resources, we need a quick method to efficiently identify whether a pedestrian is a traffic participant. Therefore, we propose a YOLO-based method to extract the ROI patch. Specifically, we first use YOLOv8 to detect the location of all traffic participation objects in the image, and then we use Intersection over Union (IoU) to identify whether the detected object is likely to participate in traffic high-risk behaviors. Finally, we cut out the maximum area that can cover these objects and then only upload the area to the cloud center.


(1) $$\begin{array}{l} IoU=\frac{BoundingBox_{A}\cap BoundingBox_{B}}{BoundingBox_{A}\cup BoundingBox_{B}} \end{array}$$

#### 3.2.2 Privacy preserving module

In this research, we expect a large number of crowdsensing participants to carry an “edge box” with the camera to sense the road environment. The visual crowdsensing paradigm of gathering public images has inevitably collided with privacy issues. Guo et al. (2017) introduced two privacy concerns: *participant privacy* and *third-person privacy*. The concern of *participant privacy* in our application is that the road sensing data uploaded by the participant while performing the task contains the participant's context, especially the GPS points. Therefore, accurate GPS points are needed for fuzzy processing. Therefore, a method is in demand for location cloaking. Our approach is to divide the urban space into different grids. After that, the edge device only uploads the grid ID of the location coordinates. The processed grid information will not reveal the specific location of the participants, and the gridding method of location cloaking will not have a great impact on the safety assessment model, as shown in Section 5.

The concerns of *third-person privacy* is the publication of potentially personally-identifiable information such as a person's face or license plate captured when gathering the road environment imagery. Zhao et al. (2022) propose a privacy-preserving MCS system called CrowdFL by seamlessly integrating federated learning (FL) into MCS. There are many works to be done in terms of privacy protection, such as privacy protection based on differential privacy mechanisms (Zhang et al., 2022; Gao et al., 2022; Wang et al., 2021; Jiang et al., 2021), using blockchain technology to ensure data privacy (Yan et al., 2022), and a distributed data storage method based on compressed sensing technology that ensures data reliability while reducing storage space and communication costs (Zhou et al., 2022), and so on. *Non-visual information extraction* in our application means image processing can be conducted in the “edge box” and only the information distilled should be delivered to the cloud center. However, the relatively powerful computing capacity of the “edge box” does not allow us to put all image processing steps on the edge side. Therefore, we prefer to use *intentional image blurring* to solve the privacy concern. Specifically, we fine-tune the YOLOv8 model deployed on the “edge box” to detect everyday traffic participation objects as well as faces and license plates. Then, the detected license plates and pedestrians' faces are blurred for privacy protection. This method is not only technically feasible but also effectively reduces the risk of privacy leakage while retaining sufficient image information for traffic behavior detection and analysis. Moreover, image blurring complies with relevant laws, regulations, and ethical standards, and is more easily understood and accepted by users, thereby enhancing participation in crowdsensing.

## 4 Traffic high-risk behavior identification framework

In this section, our goal is to identify traffic high-risk behaviors from road sensing data. Although deep learning-based traffic high-risk behavior identification has become popular and achieved excellent performance, accurately identifying various traffic high-risk behaviors in a low-cost manner remains challenging. Traditional methods require training a separate identification model for each type of traffic behavior, which is only effective for behaviors with low semantic information, such as riding without wearing a helmet. However, it is not suitable for behaviors involving multiple traffic participants, such as running a red light, due to the difficulty in labeling training data for such complex behaviors.

**Figure 4:**
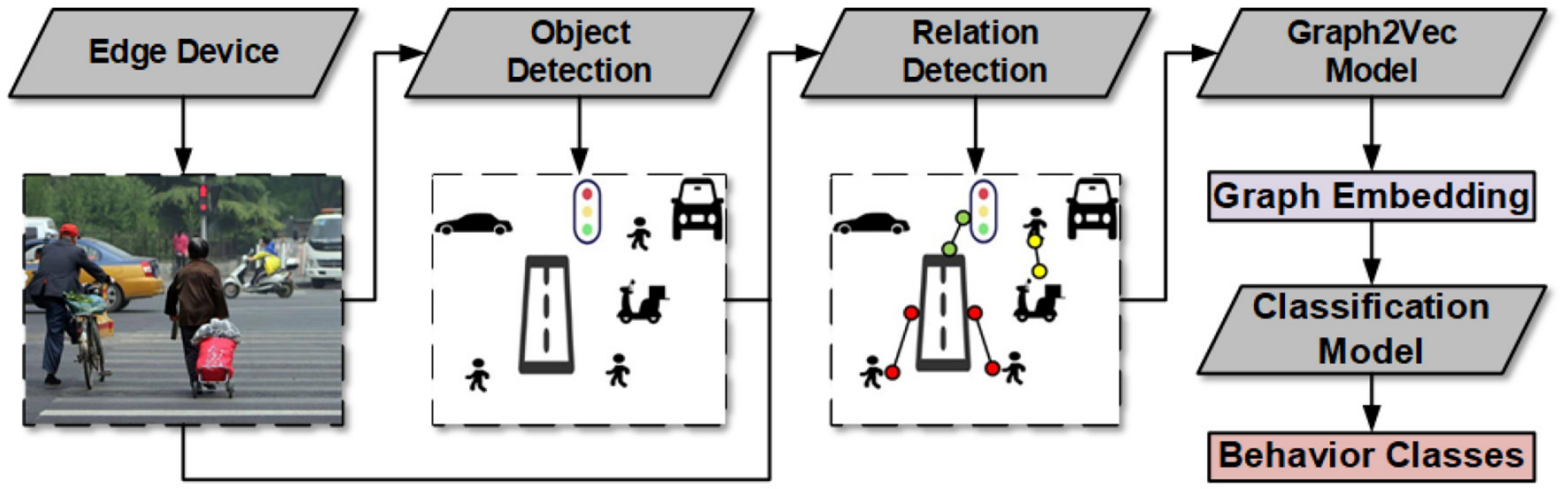
Traffic high-risk behavior detection framework overview.

In addition, we need to spend a lot of labor and non-labor resources to obtain the training data even for low semantic behaviors. Therefore, we proposed a general semantic traffic high-risk behavior detection framework as shown in Figure 4.

### 4.1 Traffic high-risk behavior detection model

In traffic high-risk behavior detection, a key issue is the identification of semantic connections between traffic participant objects. Some methods such as using deep learning networks to classify a single category (Chairat et al., 2020) are difficult to expand for other behaviors. Visual relationship detection in the field of computer vision is to detect the category and location of objects in the image, as well as the relationship between objects (Lu et al., 2016). Therefore, we introduce an object detection model to localize and classify the traffic participant objects and a relation detection model into our behavior detection framework to identify the semantic relationship between these objects.

Another key issue is how to make good use of these semantic connections. After object detection and relation detection, the image of road sensing data is transformed into a semantic graph containing only nodes and edges, in which nodes represent object information, edges represent relation information, and contain the spatial features of the objects. Intuitively, we could use a graph database like Neo4j (Webber, 2012) and then a rule-based engineering method to identify behaviors by matching the graph and manually predefined rules. Therefore, we introduce an unsupervised graph embedding framework to transfer the traffic graphs into graph embeddings. Then the graph embeddings are used for the downstream high-risk behavior classification model. The framework overview is shown in Figure 4 and the details are provided in the following section.

Firstly, we use YOLOv8 (Varghese and Sambath, 2024) to detect objects. Since crowdsensing users can use the devices we provide to collect road data in their preferred way, these road images from different perspectives result in multiple manifestations of the same object. Therefore, the traffic participation objects detection algorithms are needed to have powerful robustness. Fortunately, the rapid development of deep learning techniques (LeCun et al., 2015) has brought remarkable breakthroughs in object detection. YOLOv8 has achieved excellent performance in accuracy and speed. It has a few improvements and tricks on YOLOv4 (Bochkovskiy et al., 2020), such as mish activation and self-adversarial training, which make great progress in generalization capabilities and real-world production deployment capabilities.

Secondly, we use iCAN (Gao et al., 2018) to detect relations between traffic participant objects. The core idea of iCAN is that the appearance of objects contains informative cues for predicting relations. To exploit these cues, iCAN uses an instance-centric attention module that learns to dynamically highlight regions based on the appearance of each instance and selectively aggregate features for identifying relations. ICAN is a complete relation detection framework, including an object detection module, Faster R-CNN (Ren et al., 2015), and a relation detection module. Due to the better performance of speed and accuracy, we use YOLOv8 instead of the Faster R-CNN. Therefore, iCAN in this research refers to the relation detection module in the original iCAN framework.

Thirdly, we use graph2vec (Narayanan et al., 2017) to transfer the traffic graphs into the graph embeddings. Graph2vec is a neural embedding framework to learn data-driven distributed representations of arbitrary-sized graphs. It performs graph embedding in an unsupervised learning manner, meaning class labels of graphs are not required for learning their embeddings. This allows us to readily use graph2vec embeddings in a plethora of applications where labeled data is difficult to obtain (Narayanan et al., 2017). Furthermore, since the traffic behavior is represented as a specific graph structure in the traffic graph, the embedding obtained by graph2vec has similar characteristics if they contain the same traffic high-risk behavior.

Finally, we use a lightweight method as a behavior classification model. Through the three steps discussed previously, a traffic image was transformed into a highly refined graph embedding. This allows us to use a lightweight method to accurately classify behaviors, such as xgboost (Chen and Guestrin, 2016) or artificial neural network (ANN).

In summary, we use YOLOv8 and iCAN to abstract the semantic struct of traffic images, then an unsupervised graph2vec method is used to transfer the graph into graph embedding, which can be classified with an ANN network. Our behavior detection framework has the advantages of semantic, modular, and easily extensible categories.

### 4.2 Data labeling module

**Figure 5:**
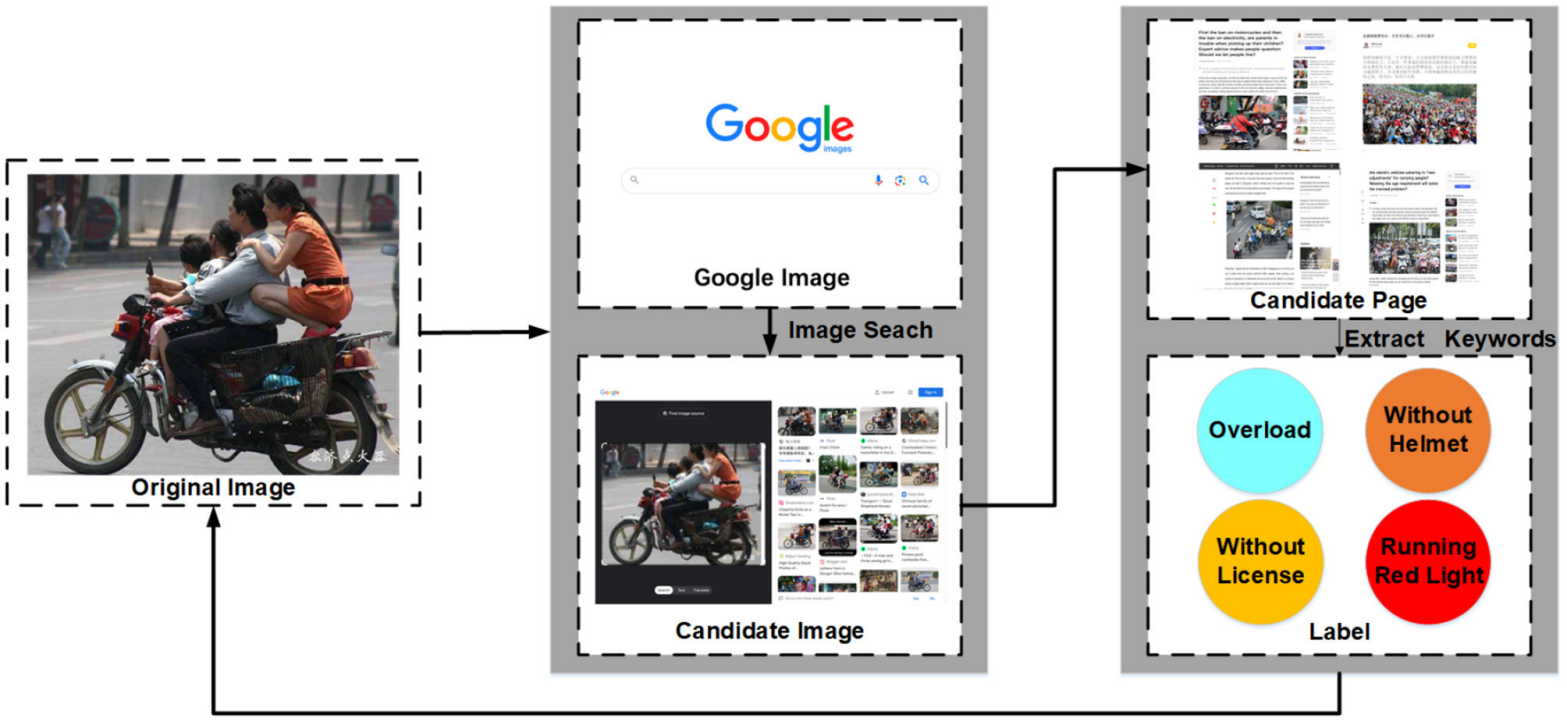
Data labeling module framework based on information retrieval.

One of the challenges of machine learning is insufficient training samples with labels (LeCun et al., 2015). In our behavior detection framework, graph2vec is an unsupervised method. Object detection and relation detection are classical problems in computer vision, so we can obtain common labels of traffic participation objects and relations. Furthermore, we consider that the process from traffic image to graph embedding can be done with a small labor cost. Therefore, we are more concerned with obtaining labels for the classification model. What we need to do is low-cost label the high-risk behaviors and collect more training samples with labels in the traffic images. As shown in Figure 5, we develop a data labeling module based on information retrieval and self-training.

Specifically, we first upload the traffic images into the visual information retrieval applications like Baidu image^1^ or Google image.^2^ Secondly, we traverse these candidate pages containing candidate images and extract keywords related to traffic high-risk behaviors. Then, these candidate images and their corresponding candidate labels are saved as candidate training samples. Finally, we will retrain the classification model in the manner of self-training (Rosenberg et al., 2005).

We manually label a dataset as the initial training data (*X*_*train*_, *y*_*train*_). In each iteration, firstly we use (*X*_*train*_, *y*_*train*_) to train a prediction model *C*_*int*_. Secondly, we collect low confident results (*X*_*lf*_, *y*_*lf*_) predicted by our models. Thirdly, crowdsensing users in our system modify and update *y*_*u*_ and add it to the training set. This iterative process does not stop until the stopping criterion is met. Algorithm 1 illustrates our self-training algorithm.

**Algorithm 1:**
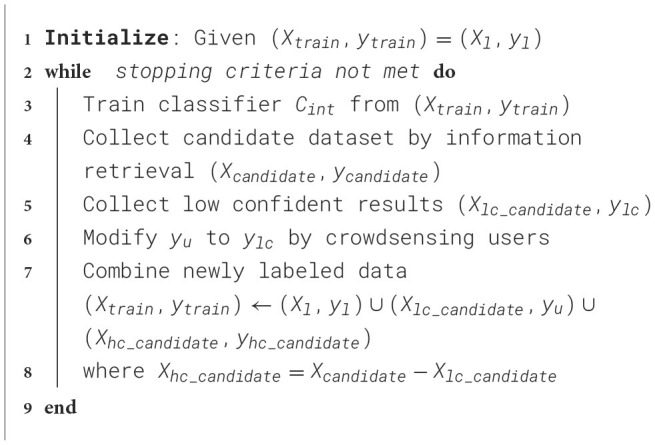
The self-training algorithm for training datasets labeling.

## 5 Urban travel safety model based on road environment data

In this section, our goal is to identify which areas in a city may have travel safety risks. First, we use urban traffic data collected from Xiamen big data open platform^3^ and Jiang et al. (2020) to compute the travel safety risk of grids. Then, we use road environment data collected through the crowdsensing paradigm to train a risk assessment model.

### 5.1 Travel safety risk computation based on urban traffic data

**Figure 6:**
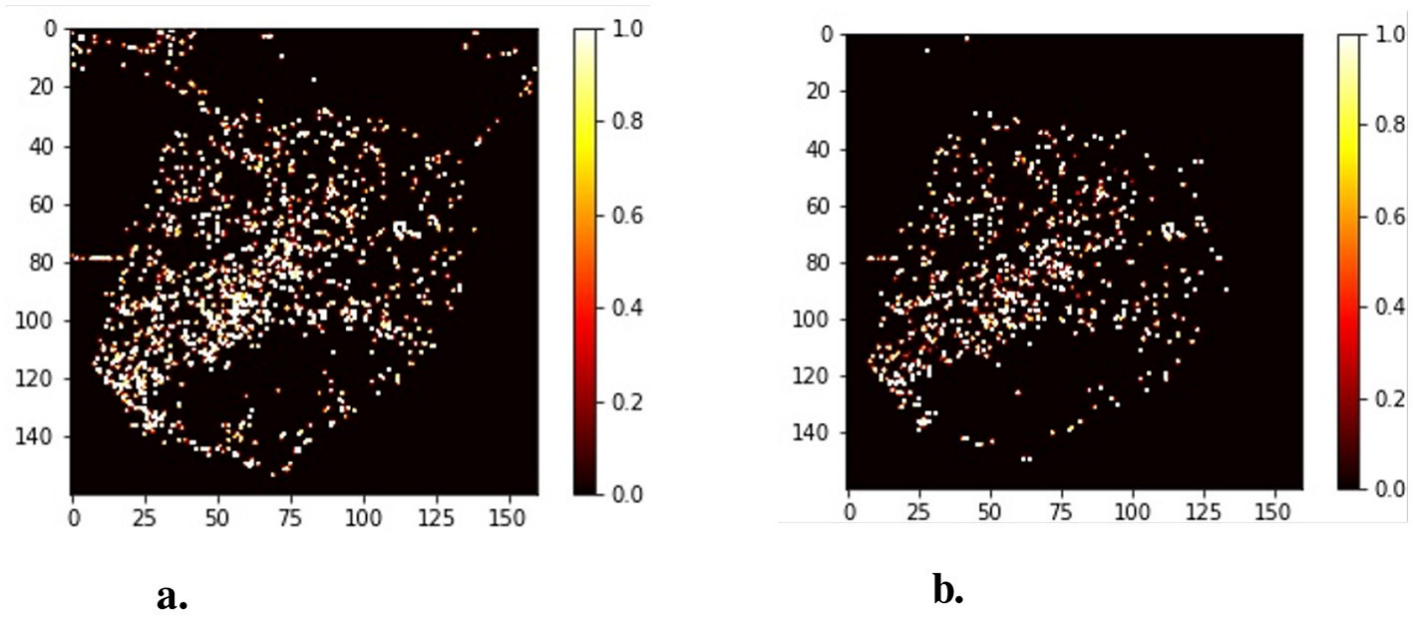
The compare of the absolute accident heatmap and the relative risk heatmap. **(A)** Traffic accidents heatmap. **(B)** Software architecture.

We collected accident data with accident details and context. Intuitively, we can directly use the number of accidents that have occurred in a grid area as a safety risk, but this method calculates the absolute risk of the grid because it does not take into account factors such as traffic flow. Therefore, a method is needed to calculate the relative risk of the grid. According to Pokorny and Pitera (2019), the truck trajectory is a key factor of traffic risk. Cyclists killed by a truck represent almost 30% of all cycling fatalities. For example, in New York City alone, 15% of bicycle networks and 11% of the truck networks are currently overlapping. Therefore, we calculate the travel safety risk of the grid based on traffic accident data and other factors such as truck flow and traffic facilities as shown in Equation 2.


(2) $$\begin{array}{l} Risk_{G}=ln\left(\frac{\overset{N}{\underset{a\subset G}{\sum }}\left(T_{a}+C_{a}\right)}{\overset{N}{\underset{t\subset G}{\sum }}N_{t}}\times e^{-\overset{N}{\underset{i\subset G}{\sum }}N_{i}}\right) \end{array}$$

where *T*_*a*_ means the traffic level of each accident and *C*_*a*_ means the context of every traffic accidents in this grid. *N*_*t*_ means the number of truck trajectories and *N*_*i*_ means the number of traffic signs in this grid *G*. The traffic flow level is categorized into three levels, with the peak period set to 1. Then, the relative travel safety risk of grids *Risk*_*G*_ is taken as the ground-truth in the risk assessment model. Figure 6 illustrates the comparison between the absolute accident heatmap (Figure 6A) and the relative risk heatmap (Figure 6B).

### 5.2 Travel safety risk modeling based on crowdsensing road environment data

According to Wu et al. (2020), the occurrence of road traffic accidents is related to pedestrians, vehicles, and road environments, such as road quality and traffic environment. We divide these influencing factors into two categories, traffic participants and road environment. For traffic participants, the most important way to affect road safety is to disobey traffic rules and conduct traffic high-risk behaviors at will. In the preceding sections, we collect road sensing data in a crowdsensing paradigm and use our proposed behavior detection framework to identify high-risk behavior. Therefore these high-risk behaviors could be covered comprehensively. However, high-risk behaviors and traffic accidents are not always positively correlated. For example, we may find a large number of high-risk behaviors in the location where the traffic flow is small and the road is wide, so although bicyclists are still flouting the rules and pedestrians are darting into traffic here, there are very few traffic accidents occurs. Therefore, we need to consider the other road environmental factors at the same time.

Road environmental factors include pedestrian flow, vehicle flow, and road facility quality (Wu et al., 2020). Intuitively, we think that the more POI there are in an area, the more people there are in that area. In addition, we use vehicle tracks as the evaluation basis of vehicle flow. The impact of road facility quality is implied in the other factors outlined previously. For example, if a region has a small flow of pedestrians and vehicles and a small number of POI, but frequent traffic accidents, it is likely that there are problems of road facility damage or unreasonable road planning in this region. Therefore, we collected urban POI data and high-risk truck behavior trajectory data as typical road environment data.

In Section 3.2, we added a random noise generated by Gaussian distribution to the user's GPS location for privacy protection. In addition, the road data have unique spatial characteristics, such as the intersection represented by crossing vehicle trajectories, which is more likely to cause traffic accidents. Therefore, we need a method to make these fuzzy coordinates not affect the location-based travel safety risk modeling and extract the spatial features.

**Figure 7:**
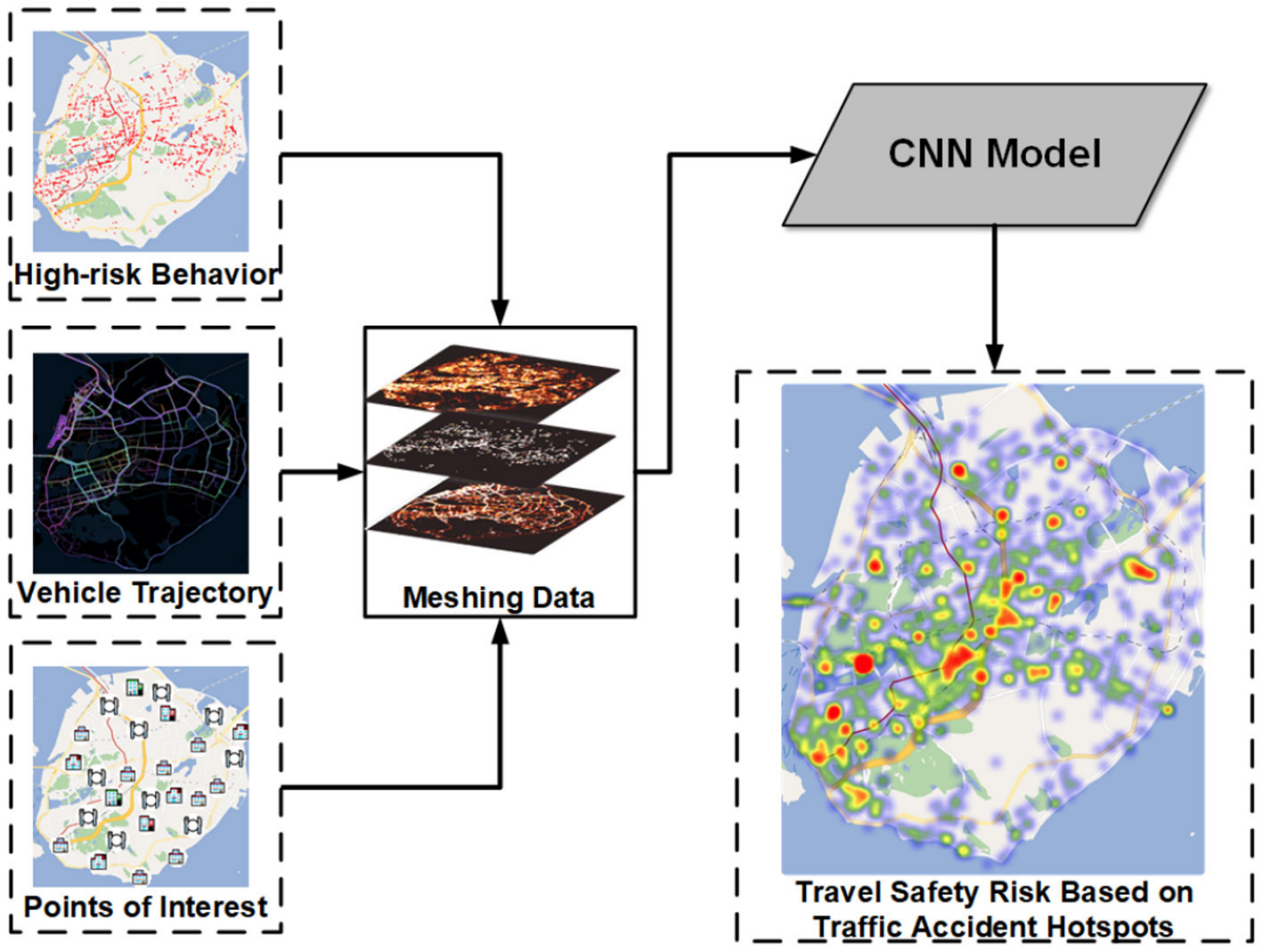
Urban travel safety model overview.

Specifically, we first map the noise-processed heterogeneous data to a unified region based on a spatial grid, where each grid covers a larger spatial area. As a result, coordinates with added noise largely remain within their original grid, minimizing any impact on the overall accuracy of risk assessment. After that, we used CNN to extract spatial features. For a region, the convolution kernel of CNN would consider both it and the surrounding region. Finally, an area of N*N size is taken as an input sample of CNN, and the travel safety risk at the sample center is used as the label to train a regression model. The risk assessment model framework is shown in Figure 7.

## 6 Evaluation

In this section, we first introduce the experiment settings and then present the evaluation results on traffic high-risk behavior detection and urban travel safety modeling. We also conduct a series of case studies to demonstrate the effectiveness of our method. Specifically, we use large-scale street view images, points of interest, and vehicle trajectories of Xiamen City as a simulation of real-world experiments to evaluate the performance. Furthermore, the details of the “edge box” prototype are shown in Table 1.

**Table 1:** Details of “edge box” prototype.

**Item**	**Description**	**Item**	**Description**
Platform	Jetson Nano	Volume	130*100*50 (mm)
Network	USB WIFI	Storage	16 GB eMMC 5.1
Camera	MIPI CSI-2	System	Ubuntu 18.04
GPS	MTK3389	JetPack	Version 4.5

### 6.1 Experiment settings

#### 6.1.1 Datasets

We evaluated our framework based on the real-world dataset collected from Xiamen. We obtain Xiamen's street views from Baidu Street View, vehicle trajectories, traffic accidents, and POI datasets. The datasets are summarized in Table 2 and the details and preprocessing steps are elaborated as follows.

**Table 2:** Summary of datasets.

**Data type**	**Item**	**Value**
Street view pictures	# Pictures	74,078
Behavior events	# Event	1,079
	Categories	4
Vehicle trajectory	# Trucks	1,304
	Sampling rate	Every minute
	Collection time	2016.9.1–2016.9.30
Points of interest	#POIs	47,439
	Categories	15
	Collection time	11/14/2017
Traffic accidents	#Accidents	100,744
	Categories	4
	Collection time	2015.1.1–2016.3.7

**Street view pictures**: street view images can help simulate the traffic risks that may be encountered in real driving scenarios (Hamim and Ukkusuri, 2024), we obtained 74,078 street view pictures in driver perspective in Xiamen from Baidu Map.^4^ These street view pictures are input into the traffic high-risk detection framework to detect traffic high-risk behaviors events, and then these events are used as one of the data sources for urban travel safety risk modeling.

**Traffic high-risk behavior events**: we obtained 1,079 behavior events of four categories detected from street view pictures in Xiamen using our high-risk behavior detection method. The selection of four categories of high-risk behavior events is based on an analysis of common high-risk behaviors for pedestrians and non-motorized traffic participants, which typically significantly increase the risk of traffic accidents (Qian et al., 2020).

**Vehicle trajectory data**: we obtained a large-scale vehicle GPS trajectory dataset from Xiamen urban big data security open platform. The vehicle dataset contains GPS trajectories of 1,304 trucks reported every 1 minute during September 2016. The rationale for selecting truck trajectory data lies in the fact that the driving trajectories of large vehicles, such as trucks, pose a higher risk to non-motorized traffic participants and pedestrians (Mehdizadeh et al., 2021).

**Points of interest**: we obtained all POIs distributions in Xiamen, which contains 47,439 POIs. We group all POIs into fifteen categories, including Hotel and hostel, Restaurant, Road and Street, Real estate, Company, Shopping, Traffic facility, Finance institution, Tourist attractions, Auto service, Business building, Life service, Entertainment, Hospital, Government agency. The selection of these categories of POI (Points of Interest) data is due to their encompassing of major crowd gathering places and facility distributions that may influence traffic flow and risk, aligning with the criteria for selecting Points of Interest in existing research (Guo et al., 2024).

**Traffic accidents**: we obtained traffic accidents in Xiamen from January 2015 to March 2016, which contain four types of accidents, including bicycle, rear-end collision, collision, and scratch, totaling 100,744 pieces. Then, we selected 4,639 accidents related to green travel (Mesimäki and Luoma, 2021).

#### 6.1.2 Evaluation metric

**Detection accuracy**: we compared the detected traffic high-risk behaviors with the ground truth dataset to evaluate the accuracy of the detection method. According to the definitions of TP, FP, and FN, we used the following indicators to quantitatively evaluate the performance of the detection method:


(3) $$\begin{array}{l} precision=\frac{\left|TP\right|}{\left|TP|+|FP\right|} \end{array}$$

(4) $$\begin{array}{l} recall=\frac{\left|TP\right|}{\left|TP|+|FN\right|} \end{array}$$

(5) $$\begin{array}{l} F1-Score=\frac{2\cdot precision\cdot recall}{precision+recall} \end{array}$$

**Travel safety modeling performance**: we use three commonly used regression model metrics to evaluate the performance of population estimation method, including RMSE (Root Mean Square Error), MAE (Mean Absolute Error) and *R*^2^ (Coefficient of Determination) score. They are defined as follows:


(6) $$RMSE=\sqrt{\frac{1}{n}\sum_{i=1}^{n}{\left(y_{i}-{\hat{y}}_{i}\right)}^{2}}$$

(7) $$MAE=\frac{1}{n}\sum_{i=1}^{n}\left|\frac{{\hat{y}}_{i}-y_{i}}{y_{i}}\right|$$

(8) $$\;R^{2}=1-\frac{\sum_{i=1}^{n}{\left(y_{i}-{\hat{y}}_{i}\right)}^{2}}{\sum_{i=1}^{n}{\left(y_{i}-\bar{y}\right)}^{2}}$$

#### 6.1.3 Baseline method

We compared our method with various baseline methods for traffic high-risk detection and the travel safety risk model. For traffic high-risk behavior detection, we compared our detection method (**YLICGV**), which uses YOLOv8 retrained using Lin et al. (2014) and the helmet dataset^5^ to detect objects and iCAN to identify relations and then use graph2vec to transfer the traffic graph into graph embedding for behavior classification, with the following baselines:

**YLIOU**: this baseline method uses a YOLO model to detect traffic participant objects and then an overlapping method to identify behaviors (Saumya et al., 2020). Specifically, we use YOLOv8 and IoU identification methods to reproduce it.

**YLGN**: this baseline method uses a YOLO model to detect traffic participant objects and then uses GoogleNet to identify behaviors (Chairat et al., 2020). Specifically, it uses YOLOv8 to detect a person, bicycle, and helmet and then train a GoogleNet to identify whether the motorcyclist is wearing a helmet.

**FRICGV**: this baseline method uses a faster R-CNN model to detect objects and iCAN to identify the relations between objects (Gao et al., 2018). Then, we use graph2vec to transfer the traffic graph into graph embedding for behavior classification. The baseline method is used to evaluate the influence of the object detection module on the performance of the overall detection framework.

The following four high-risk behaviors were taken into account and the evaluation results are shown in Tables 3, 4.

**Table 3:** Traffic high-risk behavior (overload and with helmet) detection results.

**Behaviors**	**Overload**			**Without helmet**		
**Methods**	**P**	**R**	**F1**	**P**	**R**	**F1**
YLIOU	0.917	0.930	0.923	0.941	0.901	0.921
YLGN	0.890	0.854	0.872	0.914	0.879	0.896
FRICGV	0.891	0.911	0.901	0.943	0.912	0.927
YLICGV	**0.935**	**0.945**	**0.940**	**0.955**	**0.930**	**0.942**

**P** is the precision, **R** is the recall, and **F1** is the F1-score. The bold text represents that our method is superior to other comparative methods.

**Table 4:** Traffic high-risk behavior (illegal parasol and running red light) detection results.

**Behaviors**	**Illegal parasol**			**Running red light**		
**Methods**	**P**	**R**	**F1**	**P**	**R**	**F1**
YLIOU	0.507	0.491	0.499	0.411	0.512	0.456
YLGN	0.820	0.811	0.815	-	-	-
FRICGV	0.813	0.820	0.816	0.671	0.517	0.584
YLICGV	**0.868**	**0.895**	**0.881**	**0.715**	**0.679**	**0.697**

**P** is the precision, **R** is the recall, and **F1** is the F1-Score. The bold text represents that our method is superior to other comparative methods.

*1) Bicycle overload*: A bicycle is defined as overloaded when more than two people have the relation of a person riding a bicycle.

*2) Riding without wearing helmet*: A person is defined as riding without wearing a helmet when he or she has the relation of a person riding a bicycle and has no relation to a person wearing a helmet.

*3) Illegal installation of parasol*: A bicycle is defined as installing an illegal parasol when the bicycle has the relation of a bicycle with a parasol.

*4) Running the red light*: A pedestrian or bicyclist is defined as running the red light when he or she has the relation of running zebra crossing while the traffic red light is on.

For the travel safety risk model, our method (CNN) first maps road environment datasets to a grid with a size of 160*160 after normalized processing. Then, we use the three datasets, traffic high-risk behaviors, vehicle trajectories, and POI in Xiamen, as the different channels of the input dataset. Thirdly, the size of 5*5 grids was taken as a sample and the traffic accident risk of the center in the sample was taken as the label. Finally, we use the CNN model to model the travel risk. We compared our method with the following baselines.

**RF**: this baseline method used traffic high-risk behaviors, vehicle trajectories, and POIs as the input dataset. Specifically, the size of 5 × 5 grids was flattened and used as an input sample to train a random forest model.

**ANN**: this baseline method used traffic high-risk behaviors, vehicle trajectories, and POIs as the input dataset. Additionally, the size of 5 × 5 grids was flattened and used as an input sample to train an artificial neural network model.

**CNN-PT**: this baseline method used POIs and vehicle trajectories as the input dataset. Moreover, the size of 5 × 5 grids was used as an input sample to train a convolutional neural network model.

**CNN-BT**: this baseline method used traffic high-risk behaviors and vehicle trajectories as the input dataset. Furthermore, the size of 5 × 5 grids was used as an input sample to train a convolutional neural network model.

### 6.2 Experiment results

#### 6.2.1 Traffic high-risk behavior detection results

**Figure 8:**
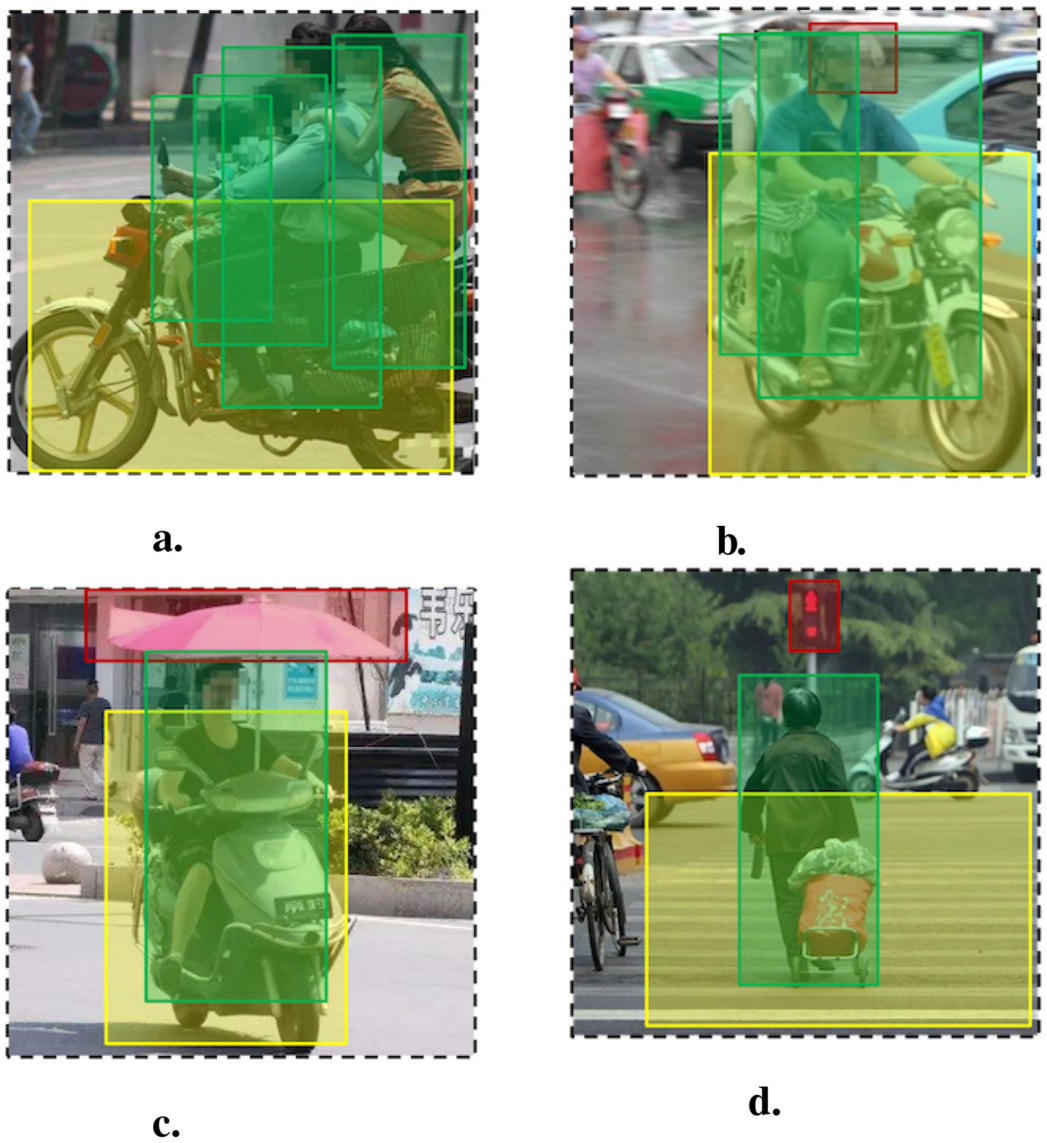
Traffic high-risk behavior detection result examples. **(A)** Overload. **(B)** Wearing helmet. **(C)** Illegal parasol. **(D)** Running red light.

We compare the overall accuracy of different methods for the four behaviors discussed in Tables 3, 4. We can see that our **YLICGV** method achieves the best overall accuracy, outperforming the other baseline methods. As shown in Figures 8A, B, the **YLIOU** method performed close to or even better than our method in the categories of without helmet and overload, but poorly in the categories of illegal installation of parasol shown in Figure 8C, in which parasol and person have low IoU rate, and running the red light shown in Figure 8D, in which has more traffic objects. The reason is that **YLIOU** applies to the low-semantic behaviors in which traffic objects have significant spatial coverage features.

In addition, the **YLGN** shows relatively average accuracy, but it could not identify the behavior of running the red light. It is difficult to obtain labeling data as this highly semantic behavior involves multiple objects in a complex scenario. Furthermore, the **FRICGV** achieves relatively high accuracy for detecting four behaviors. It shows that using a relation detection model and graph2vec model could detect both low-semantic and high-semantic behaviors. Moreover, employing a better object detection model such as YOLOv8, instead of RCNN, would further improve the performance of the detection framework.

#### 6.2.2 Travel safety risk model results

**Figure 9:**
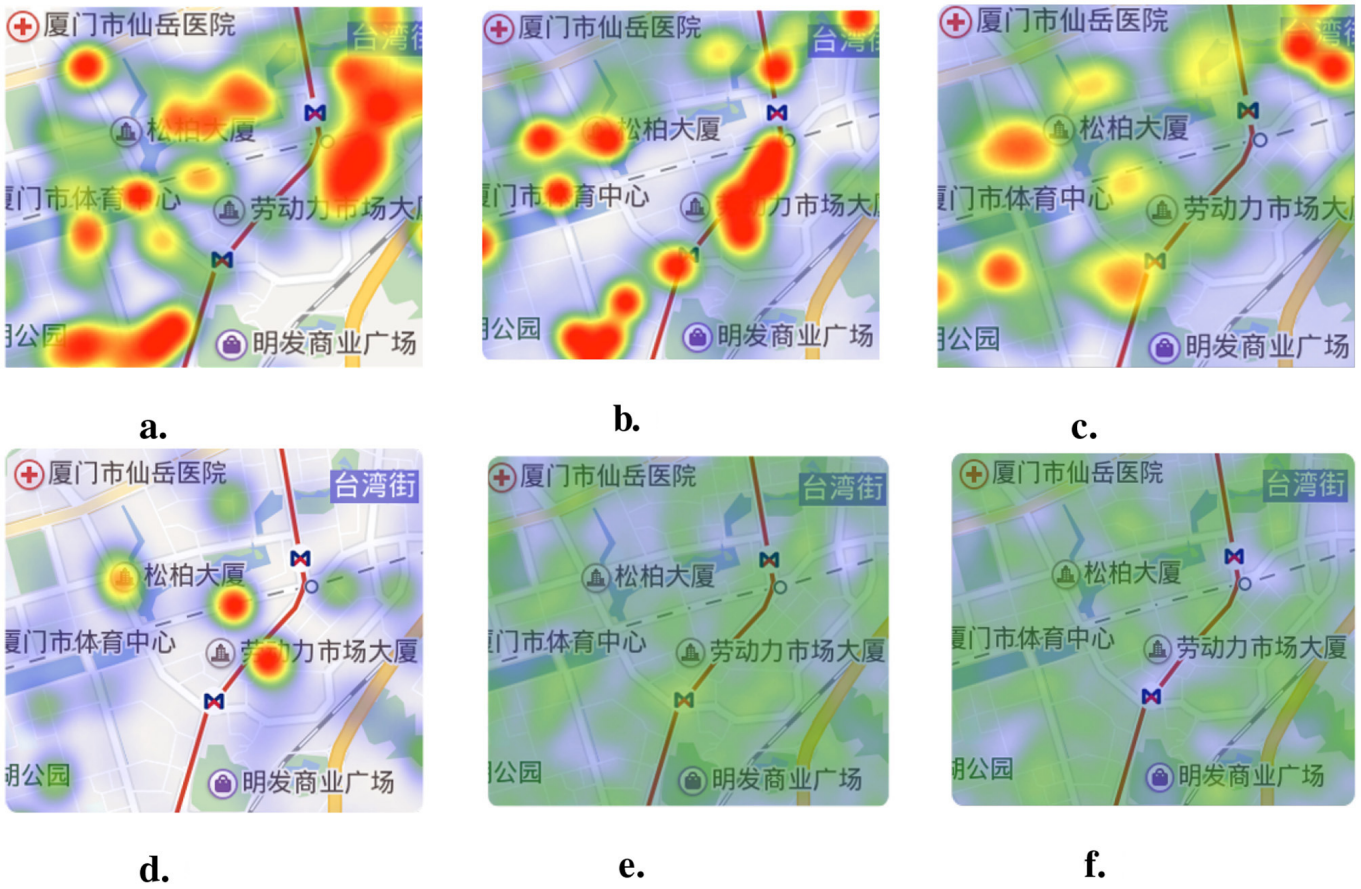
Urban travel safety risk assessment result examples. **(A)** Ground truth. **(B)** CNN result. **(C)** CNN-BT result. **(D)** CNN-PT result. **(E)** ANN result. **(F)** RF result.

We present the risk assessment result in Table 5. It shows that the **CNN** method achieves the best performance among all the baselines. The **RF** and **ANN** models perform the worst assessment accuracy since the flattened data loses its spatial features. The assessment examples shown in Figures 9E, F shows **RF** and **ANN** model assess the travel risk in a smoother manner. In addition, the **CNN-PT** and **CNN-BT** achieve the relatively high performance. It shows that the CNN model with spatial feature extraction capability is suitable for this scenario and concatenating traffic high-risk behaviors and the other single road environment data is not enough for building an effective assessment model. Furthermore, the better performance of **CNN-BT** than **CNN-PT** shows that traffic risk behaviors have a greater impact on assessment performance. Our **CNN** method further improves the performance by leveraging multi-source road environment data and convolutional kernels to effectively fuse three types of features, achieving R^2^ of 0.85, outperforming the other baseline methods. The comparison of experimental results is shown in the Figure 9.

**Table 5:** Travel safety risk model results.

**Methods**	**RMSE**	**MAE**	**R^2^**
RF	0.1693	0.0230	0.23
ANN	0.1631	0.0216	0.25
CNN-PT	0.1623	0.0220	0.36
CNN-BT	0.1615	0.0217	0.43
CNN	**0.1251**	**0.0133**	**0.85**

The bold text represents that our method is superior to other comparative methods.

### 6.3 Case studies

We conducted case studies on travel safety risk evaluation on Xiamen Island. First, by comparing the ablation results of risk assessment models, we demonstrated the importance of urban crowdsensing datasets, specifically traffic high-risk behaviors detected from our behavior detection framework. Then, we demonstrated the effectiveness of our risk assessment model by comparing the real traffic accident data with the predicted results of the risk assessment model.

#### 6.3.1 Lingdou Community to Software Park

**Figure 10:**
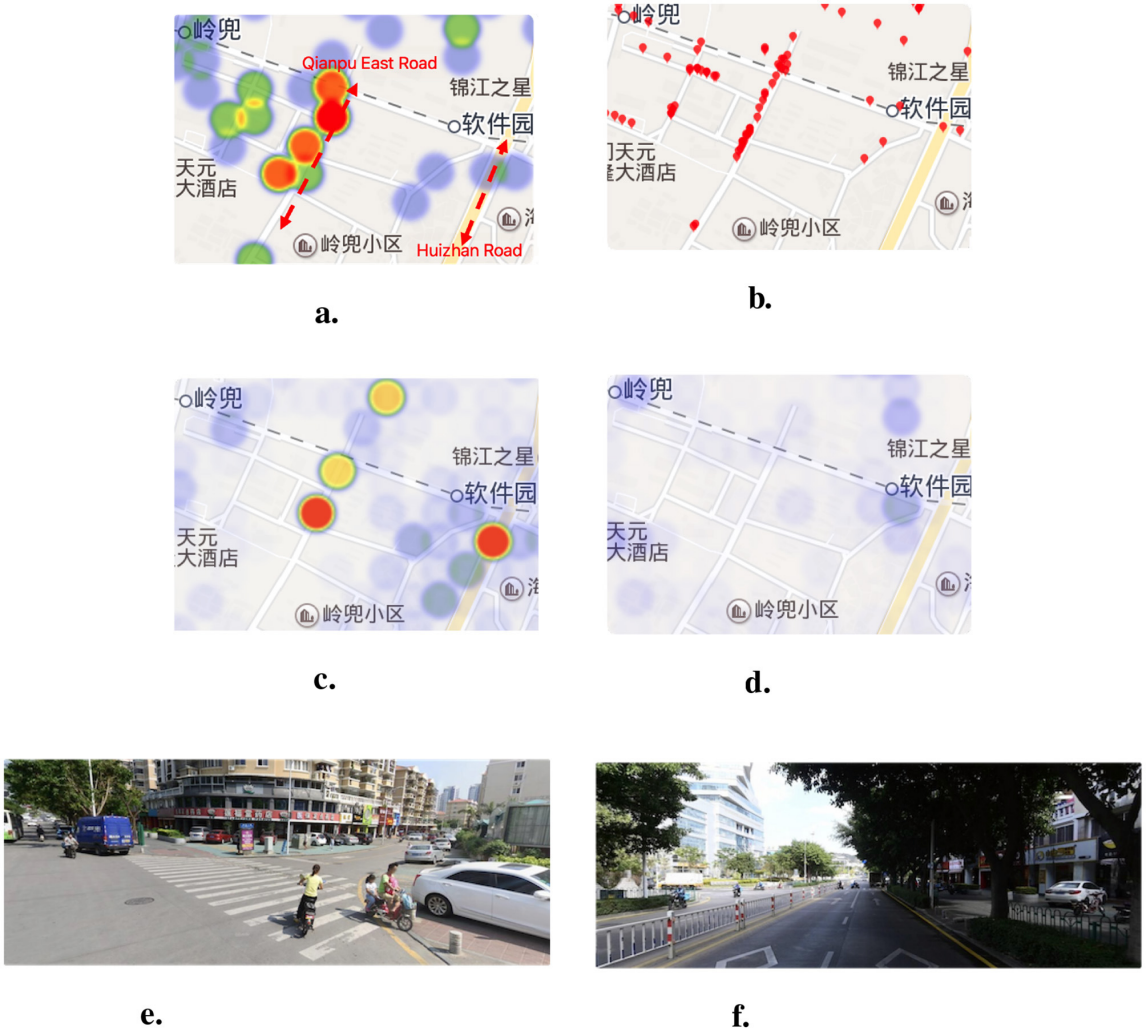
The comparison of high-risk points and behaviors of Qianpu East Road and Huizhan Road from Lingdou Community to Software Park. **(A)** The high-risk points from traffic accidents. **(B)** The high-risk behaviors detected by our framework. **(C)** The predicted high-risk points by CNN model. **(D)** The predicted high-risk points by CNN-PT model. **(E)** The street view of Lingdou Community in 2019. **(F)** The street view of Software Park in 2019.

Figure 10 illustrates the case study at Lingdou. Figures 10A, B show respectively the high-risk points and high-risk behaviors of Qianpu East Road and Huizhan Road near to Lingdou Community. We can see that the distribution of high-risk points from real traffic accidents is similar to the distribution of the high-risk behavior events to a large extent. This indicates that traffic high-risk behavior data plays an important role in travel safety risk assessment. Figure 10C shows that the CNN model predicts Qianpu East Road and Huizhan Road as high-risk points. However, as shown in Figure 10D, the CNN-PT model does not take into account the data on traffic high-risk behaviors, so it only predicts Huizhan Road as a medium-risk point. The case demonstrates that our method is essential for risk assessment to detect traffic high-risk behaviors. In addition, Lingdou Community and Qianpu are large urban villages in which people are complicated and have weak traffic safety awareness. Some traffic high-risk behaviors such as overloading and riding without wearing safety helmets are shown in Figures 10E, F.

#### 6.3.2 Zhenhai Road station to Lujiang Road

**Figure 11:**
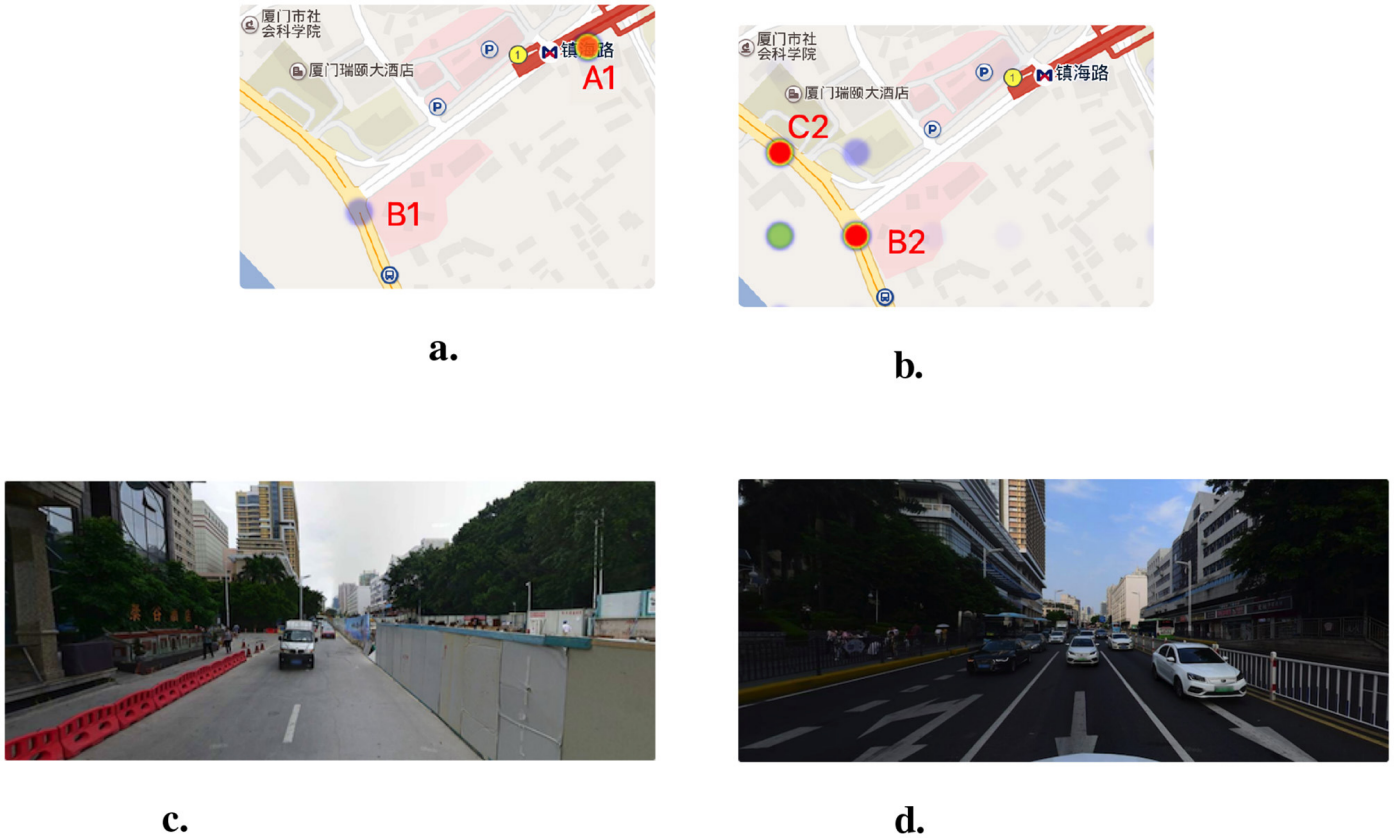
The comparison of high-risk points in Zhenhai Road Station and street view images in different time. **(A)** The high-risk points from traffic accidents during 2015–2016. **(B)** The predicted high-risk points by our model. **(C)** The Zhenhai Road Station in 2015. **(D)** The Zhenhai Road Station in 2019.

From 2015 to 2016, traffic accidents data showed that the Zhenhai Road station was a high-risk point (A1, as shown in Figure 11A was a medium-risk point (B1 as shown in Figure 11A) because the road was under construction during this period. Construction facilities such as construction site barriers (shown in Figure 11C) make bicycles and pedestrians crowded along the road, so bicycle accidents at the A1 location were frequent. In addition, the risk assessment model trained with the urban crowdsensing data predicts that the location of A1 was not a high-risk point. This is because, after the completion of the subway construction, the traffic department set up railings to separate the motor vehicle lanes and the sidewalks (shown in Figure 11D). Therefore, the A1 point where the subway station was located has a low accident risk, and the intersections near the subway station, namely the B2 and C2 points (shown in Figure 11B), have a high accident risk. In September 2018, the analysis presented was verified by a news report that an e-bike and an earth-moving vehicle accident at B2 resulted in the injury of an e-bike driver and the death of a backseat rider.^6^ The case demonstrates the lag in assessing travel risk based solely on historical accident data and the effectiveness of our risk assessment method.

## 7 Related work

In this section, we first introduce mobile crowdsensing and then present an overview of traffic behavior detection and traffic safety risk modeling.

### 7.1 Mobile crowdsensing

Mobile crowdsensing has recently emerged as a popular mobile-user-centric paradigm that leverages ubiquitous mobile devices to enable large-scale applications. As sensor-rich mobile devices become more prevalent, mobile crowdsensing has proven to be a cost-effective sensing paradigm. Mobile crowdsensing applications have emerged in the environment, infrastructure, and society in recent years. For example, Dutta et al. (2009) developed a participatory sensing system, Common Sense, which uses handheld air quality sensing devices that communicate with mobile phones to measure various air pollutants. Mobile crowdsensing applications in infrastructure include measuring traffic congestion, road conditions, traffic prediction, etc. For example, Tong et al. (2022) leveraged the phone's built-in sensors to locate and navigate vehicles in urban areas where satellite coverage was limited or unavailable, such as large-scale tunnels, underground parking lots, and multilevel flyovers. Furthermore, Wang et al. (2022) proposed a spatiotemporal urban inference and prediction framework based on sparse mobile crowdsensing, which was used to predict future traffic congestion and parking occupancy in cities. The third category is social applications such as Ubigreen (Froehlich et al., 2009), which encourages green transportation by semi-automatically tracking transit activity in the form of smartphone sensing and user self-reported.

However, existing research primarily focuses on environmental factors, infrastructure monitoring, or traffic prediction, with limited attention to high-risk traffic behaviors, which are critical factors in assessing traffic safety. Moreover, while mobile crowdsensing has been used for various traffic-related applications, few studies have directly addressed the risk of traffic accidents by considering the complex interactions of road users' behaviors and environmental factors. In this research, we proposed a high-risk behavior identification and traffic risk modeling framework based on mobile crowdsensing to assess urban traffic green travel safety.

### 7.2 Traffic high-risk behavior detection

The fatalities caused by bicyclist and pedestrian traffic high-risk behaviors have doubled in recent years in the United States (National Highway Traffic Safety Administration, 2013) and China.^7^ Bicyclists and pedestrians are more vulnerable to injury in traffic conflicts compared with motor vehicles. The bicyclist and pedestrian traffic high-risk behavior detection problem has been studied by many researchers using different sensors and computer vision algorithms. Ooi et al. (2016) developed a detection system for bicyclist traffic high-risk behaviors, using Kinect's skeleton tracking function and illuminance sensors. Tanaka and Takami (2018) developed a smartphone application to detect cyclists' high-risk behaviors of stop sign rules using smartphone sensors, aiming to supplement safe bicycle ride education at home. For pedestrian high-risk behaviors detection, Zaki and Sayed (2014) focused on pedestrian high-risk behaviors in intersections using computer vision algorithms, such as object tracking, to analyze pedestrian behaviors like pedestrians crossing the red light. Špaňhel et al. (2018) used the deep learning-based object detection algorithm Faster R-CNN to detect road users in intersections for detecting their traffic high-risk behaviors and deployed their system in the NVIDIA Jetson platform.

However, few studies have been conducted on traffic high-risk behavior detection using mobile edge devices, which limits the scalability of such systems for large-scale traffic risk sensing. In contrast, our approach leverages the power of mobile crowdsensing and edge computing, enabling real-time, large-scale detection and continuous monitoring of high-risk traffic behaviors across urban environments. This allows for the integration of data from a wide range of mobile devices, significantly enhancing the coverage and effectiveness of traffic safety risk assessments.

### 7.3 Traffic accident risk assessment

Owing to the availability of large volumes of traffic flow data along with traffic accident information, there is a renewed opportunity for the development of models for the assessment or prediction of traffic accident risk. The current methods model the relationship between the complex traffic accident context and traffic accident risk by manual construction features or machine learning models. For example, Lin et al. (2015) proposed a Frequent Pattern tree-based variable selection method to identify all the frequent patterns in the traffic accident dataset and then calculate the importance score of each explanatory variable. Ren et al. (2017)collected heterogeneous traffic-related data, including traffic accidents, traffic flow, weather conditions, and air pollution from the same city; proposed a deep learning model based on a recurrent neural network toward a prediction of traffic accident risk. In addition, Yuan et al. (2018), Park et al. (2016), and Wenqi et al. (2017) directly predict traffic accidents by deep learning methods based on traffic accident-related datasets.

In this research, we argue that traffic accidents, as random events, cannot be directly predicted. Instead, the risk of accidents should be continuously assessed. Existing methods often overlook key factors like traffic high-risk behaviors, which significantly contribute to accident likelihood. Therefore, we propose a traffic safety risk assessment model using CNN that integrates crowdsensing data, including high-risk behaviors and truck trajectories, to provide a comprehensive and real-time risk assessment.

## 8 Discussion

We discuss the following limitations of our framework.

**(1) Identifying more diverse traffic high-risk behaviors**. In this research, we focus on the traffic behaviors of urban residents while green travel. This is not only because green travel has become the preferred mode of travel for more and more people, but also because the traffic high-risk behaviors during green travel have obvious human-centered characteristics. This allows us to obtain annotated data more easily. However, our semantic behavior detection framework can be applied to more diverse traffic behaviors. Therefore, we believe that our semantic recognition framework will have further application with more human resources and the development of relation detection technology.

**(2) Further privacy-preserving for third-person and participants**. In this research, we required some traffic participants equipped with the “edge box” to collect the road sensing data. In this process, the identities and locations of users, and the sensing data of users will all be obtained by the platform and disclosed to third parties when the platform is untrusted (Yu et al., 2017). Therefore, we use a data preprocessing module to protect users' privacy by blurring persons' face and plate licenses and adding Gaussian noise to location coordinates. However, the method still exposes partial sensitive data and approximate location. In the future, we plan to introduce personalized local differential privacy (LDP)-based privacy-preserving data collection.

**(3) Travel safety risk assessment based on spatial-temporal data**. In this research, we mainly focus on the spatial features in traffic behaviors and road environment datasets in safety risk assessment. The reason is that the temporal dimensions of different datasets are not uniform. For example, our detection framework's traffic behaviors detected from street view images lack a temporal dimension. In addition, vehicle trajectories and traffic accidents were collected at different periods. In the future, we consider the actual deployment of edge devices to collect Spatio-temporal data and then use some Spatio-temporal algorithms like STGCN (Wang et al., 2019) to optimize our method. Furthermore, our current work does not account for the impact of a traffic participant who frequently engages in high-risk traffic behavior on the framework.

## 9 Conclusion

In this paper, we proposed a mobile edge crowdsensing framework to dynamically assess urban traffic green travel safety risk, which could reduce the labor and non-labor cost to continuously collect road sensing data and detect traffic high-risk behaviors. In terms of engineering design, we developed a low-cost, high-performance edge device based on Nvidia Jetson Nano, which can efficiently and securely collect road sensing data. In the traffic high-risk behavior detection framework, we propose an accurate, semantic, and general model based on object detection, relation detection, and the graph2vec model to accurately and cost-effectively identify traffic high-risk behaviors. Additionally, in terms of data labeling, we developed a data labeling module based on visual information retrieval and self-training methods. In the travel safety assessment model, we collected multi-source and heterogeneous urban crowdsensing road environment data to collaboratively build the safety assessment model. Our framework achieves an average F1-score of 86.5% in accurately detecting traffic high-risk behaviors and an average *R*^2^ value of 0.85 for assessing travel safety risks, outperforming various baseline methods. In terms of urban traffic management, real-world experiments conducted in Xiamen Island further validated its effectiveness.

In the future, we plan to refine and deploy edge devices, explore more complicated traffic behaviors and incorporate Spatio-temporal road environment data to further improve the performance of the travel safety risk assessment. We will also work with local governments or transportation departments to use existing road monitoring data as input to promptly intervene or punish violators after identifying risks.

## Data Availability

The original contributions presented in the study are included in the article/supplementary material, further inquiries can be directed to the corresponding author.

## Footnotes

^1^ https://image.baidu.com/

^2^ https://images.google.com/

^3^ https://data.xm.gov.cn/opendata/index.html

^4^ https://map.baidu.com

^5^ https://github.com/njvisionpower/Safety-Helmet-Wearing-Dataset

^6^ https://weibo.com/1778455640/GAgQaqjn8

^7^ http://www.stats.gov.cn/

## Appendix

The translations of the Chinese place names on the maps in this article are as follows:

**Table A1:** Translation of chinese street names.

**Label**	**Chinese street name**	**English street name**
Figures 9A–F	厦门市仙岳医院	Xiamen Xianyue Hospital
Figures 9A–F	松柏大厦	Songbai Building
Figures 9A–F	厦门市体育中心	Xiamen Sports Center
Figures 9A–F	劳动力市场大厦	Labor Market Building
Figures 9A–F	明发商业广场	Minfa Business Plaza
Figures 9A–F	台湾街	Taiwan Street
Figures 10A–D	岭兜	Lingdou
Figures 10A–D	天元大酒店	Tianyuan Hotel
Figures 10A–D	岭兜小区	Lingdou Community
Figures 10A–D	锦江之星	Jinjiang Inn
Figures 10A–D	软件园	Software Park
Figures 11A, B	厦门市社会科学院	Xiamen Academy of Social Sciences
Figures 11A, B	厦门瑞颐大酒店	Swiss Grand Xiamen
Figures 11A, B	镇海路	Zhenhai Road
